# Ecological and Evolutionary Determinants of Bark Beetle —Fungus Symbioses

**DOI:** 10.3390/insects3010339

**Published:** 2012-03-22

**Authors:** Diana L. Six

**Affiliations:** Department of Ecosystem and Conservation Sciences, College of Forestry and Conservation, University of Montana, Missoula, MT 59812, USA; E-Mail: diana.six@cfc.umt.edu

**Keywords:** *Ophiostoma*, *Grosmannia*, *Leptographium*, *Ceratocystiopsis*, *Ceratocystis*, *Raffaelea*, *Ambrosiella*, cospeciation, host-switching, symbiosis, symbiont redundancy, ambrosia beetle

## Abstract

Ectosymbioses among bark beetles (Curculionidae, Scolytinae) and fungi (primarily ophiostomatoid Ascomycetes) are widespread and diverse. Associations range from mutualistic to commensal, and from facultative to obligate. Some fungi are highly specific and associated only with a single beetle species, while others can be associated with many. In addition, most of these symbioses are multipartite, with the host beetle associated with two or more consistent partners. Mycangia, structures of the beetle integument that function in fungal transport, have evolved numerous times in the Scolytinae. The evolution of such complex, specialized structures indicates a high degree of mutual dependence among the beetles and their fungal partners. Unfortunately, the processes that shaped current day beetle-fungus symbioses remain poorly understood. Phylogeny, the degree and type of dependence on partners, mode of transmission of symbionts (vertical *vs.* horizontal), effects of the abiotic environment, and interactions among symbionts themselves or with other members of the biotic community, all play important roles in determining the composition, fidelity, and longevity of associations between beetles and their fungal associates. In this review, I provide an overview of these associations and discuss how evolution and ecological processes acted in concert to shape these fascinating, complex symbioses.

## 1. Scolytinae-Fungus Symbioses

The term symbiosis was coined by Albert Frank in 1877 to describe nonparasitic interactions involving microbes [1]. The meaning was further refined by de Bary in 1879 to mean “the living together of two differently named organisms” [1], a definition that remains in widespread use today. Symbioses encompass a wide range of interaction types. Among the least studied are mutualisms, once relegated to the status of curiosities of nature, but now considered important determinants of biological organization, and community structure and process [2,3,4,5]. In this review, I consider several factors that may have shaped a diverse array of ectosymbioses, including mutualisms, among bark beetles and fungi. For more general treatments of these symbioses, I refer the reader to several recent reviews [6,7,8,9,10,11,12]. In the context of scolytine beetle-fungus interactions, both the beetle and the tree they infest are often referred to as hosts. To avoid confusion, I will confine my use of the term “host” in this chapter to denote strictly the beetle.

Bark beetles make up approximately 3700 of the 7500 species in the weevil (Curculionidae) subfamily Scolytinae [13,14,15]. The remainder consists of ambrosia beetles (3400 species) and various seed and pith-feeding beetles (~400 species). A striking characteristic of the Scolytinae is the widespread association of its members with fungi. All ambrosia beetles, and many bark beetles, are associated with fungi [7,9,16]. Of the seed and pith feeders, little is known. However, fungi are associated with members of this group as diverse as conifer cone beetles (*Conophthorus* spp.) (Six, pers. obs.) and the coffee berry borer (*Hypothenemus hampei*) [17].

Bark beetles are commonly associated with Ascomycetes in four teleomorph genera, *Ophiostoma*, *Ceratocystiopsis*, *Grosmannia*, and *Ceratocystis *[7,9,10,18]. While these fungi produce morphologically similar teleomorphs, *Ophiostoma*, *Grosmannia*, and *Ceratocystiopsis* form a monophyletic group in the Ophiostomatales, separate from *Ceratocystis*, which is inthe Microascales [19,20]. The two fungal groups also have different host plant affiliations. The fungi in the Ophiostomatales are most often associated with conifers, while *Ceratocystis* species are usually associated with angiosperms [21]. Anamorphs associated with *Ophiostoma* and *Ceratocystiopsis* include *Hyalorhinocladiella* and *Sporothrix*, while some *Ophiostoma* species also produce *Pesotum*. *Grosmannia* species produce *Leptographium* anamorphs [18], whereas *Ceratocystis* produce *Thielaviopsis* anamorphs [22]. A relatively small number of bark beetles are consistently associated with Basidiomycetes in the genera *Entomocorticium *and *Phlebiopsis* [23,24].

Ambrosia beetles are often associated with anamorphic species in the genera *Ambrosiella *and *Raffaelea* but some are also associated with *Ophiostoma*, *Leptographium*, and *Fusarium *[9,16,25,26,27,28]. Interestingly, early molecular phylogenies revealed that *Ambrosiella* and *Raffaelea* were each paraphyletic and multiply derived out of *Ophiostoma* and *Ceratocystis *[29,30]. Furthermore, one monospecific genus *Dryadomyces* was found to nest within a clade containing both *Ambrosiella* and *Raffaelea* species allied with *Ophiostoma* [31]. These inconsistencies were addressed by Harrington *et al. *[32] who retained all *Ambrosiella* with *Ceratocystis* affinities within *Ambrosiella* but transferred those associated with the Ophiostomatales to *Hyalorhinocladiella*. New combinations were made in *Raffaelea* for *Ambrosiella* species allied with the Ophiostomatales as well as a transfer of *Dryadomyces* to *Raffaelea*. 

**Figure 1:**
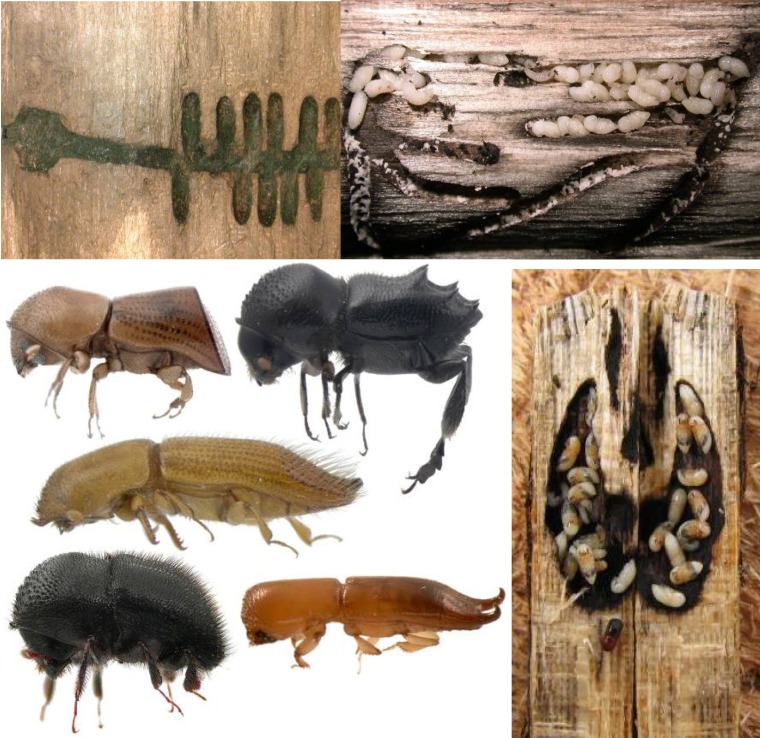
Examples of ambrosia beetles and their galleries. From left to right, top to bottom: *Diuncus* gallery; *Trypodendron* gallery; Xyleborina ambrosia beetles; *Xylosandrus crassiusculus* gallery. All photos courtesy of Jiri Hulcr.

**Figure 2:**
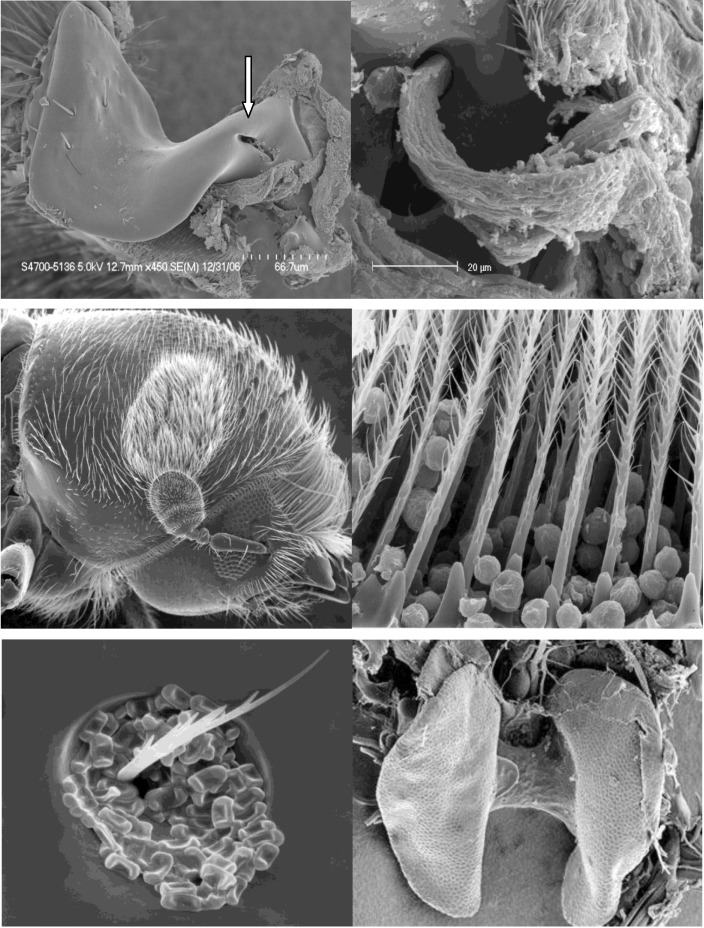
Examples of mycangia. From left to right, top to bottom: maxillary cardine of *Dendroctonus ponderosae* showing opening of sac mycangium (arrow) courtesy of Katherine Bleiker; Close up of mycangium of *D. ponderosae* showing fungal mass extruding from opening courtesy of Katherine Bleiker; Oval brush mycangium on female *Pityoborus rubentris* Mal Furniss; close up of brush mycangium of *P. rubentris* containing spores Mal Furniss; Ascospores in pit mycangium (puncture) of *Ips pini* Mal Furniss; mesonotal paired sac mycangia of *Xylosandrus mutilates* (dissected from beetle) courtesy of W. Doug Stone.

Bark and ambrosia beetles are categorized by their use of host plant substrate, but there is no absolute distinction between the two groups and most are associated with fungi to some extent. Most ambrosia beetles construct galleries in the sapwood of trees (Figure 1). The term ‘ambrosia’ refers to the fungal gardens the beetles cultivate on their gallery walls and use as an exclusive food source [16,33]. The beetles are obligately dependent upon the fungi, from which they acquire amino acids, vitamins and sterols [16,33]. The activities of female beetles have been hypothesized to control the growth and composition of ambrosial gardens. If the female dies, the garden is quickly overgrown by contaminating fungi and bacteria, which ultimately results in the death of the brood [26,34]. The activities of the larvae may also control non-mutualistic fungi, although the mechanism for this is unknown (X). Dispersing adult beetles transport the fungi to new host trees in highly specialized structures of the exoskeleton called mycangia (Figure 2), thus maintaining the association from generation to generation [7,35]. The interaction is clearly mutualistic. The symbiosis allows the beetles to exploit a nutritionally poor resource (wood) and reduce interspecific competition, while providing the fungi consistent transport to a relatively rare and ephemeral resource (a new host tree of the appropriate condition and successional stage) [11,16].

**Figure 3:**
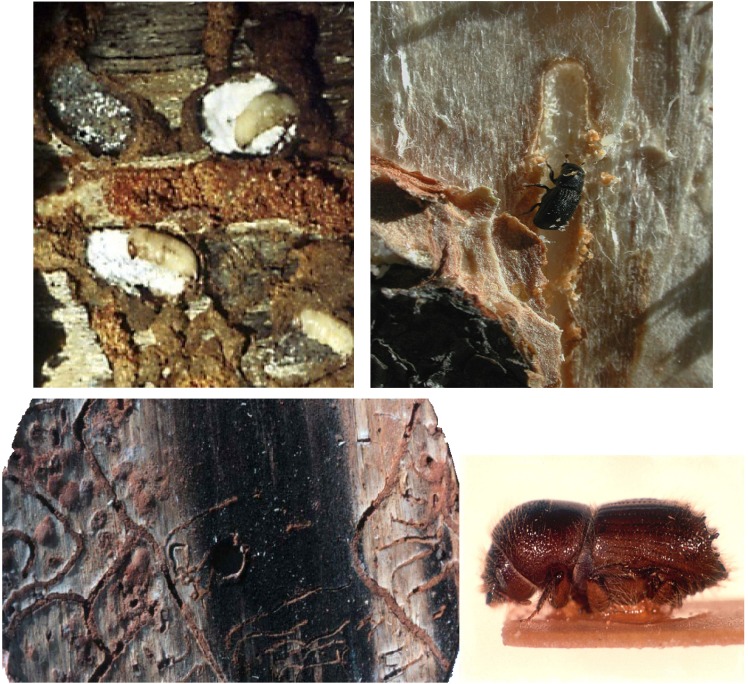
Examples of bark beetles and their galleries. Left to right, top to bottom: Pupal chambers containing *Dendroctonus.ponderosae* pupae and spore layers of fungi, courtesy of the author; *D. ponderosae* adult, courtesy of the author; Bark section showing extensive beetle development in light portions colonized by mutualistic fungal symbionts and lack of development in highly stained portion of bark colonized by the antagonistic fungus, *O. minus* (arrow) courtesy of Fred Stephen; *I. pini* courtesy of Jesse Logan.

In contrast, bark beetles construct their galleries in the phloem layer of trees just under the outer bark (Figure 3). Unlike ambrosia beetles, bark beetles feed on tree tissues (phloem), and gain some of their nutrients directly from the host. Phloem contains more nutrients than sapwood, but nonetheless has a low nutritional value relative to the dietary requirements of insects [36,37,38]. Nitrogen is the limiting factor in the diets of most herbivorous insects [39]. This is true even for insects that feed on foliage, which is relatively high in nitrogen compared with other tree tissues, including phloem. For instance, the nitrogen content of loblolly pine phloem (a host to several bark beetles) is approximately 0.38% [40] compared with 1–5% in the foliage [39]. Insects contain approximately 6–10% nitrogen, indicating that to complete development they must either consume large amounts of plant material relative to their final body size [41,42] or modify their diet in such a way as to increase the nitrogen content [38]. In the case of bark beetles, diet modification may include the use of fungal associates to supplement the nutritional limitations of their phloem diet [38,43].

Evidence supports the existence of both high consumption and diet modification strategies in bark beetles. Ayres *et al.* [38] compared nitrogen budgets of two co-occurring bark beetles, *Ips grandicollis* and *D. frontalis*, which have different feeding strategies. *Ips grandicollis* is a non-mycangial beetle that constructs long feeding galleries in phloem. In contrast, *Dendroctonus frontalis*, a mycangial beetle, produces short galleries terminating in ‘feeding chambers’ where it spends most of its development feeding on ambrosial growth of its mycangial fungi [44, S.J. Barras, pers. comm.]. Ayres *et al.* [38] found that the nitrogen concentration around successfully developing larvae of *D. frontalis* is more than twice that of phloem of uninfested trees; the phloem with the highest nitrogen concentration was located where feeding chambers were colonized by the mycangial fungi. Similarly, Hodges *et al.* [43] also found that phloem nitrogen in *Pinus taeda* increased 131% when *D. frontalis* and its associated fungi were introduced.

Ayres *et al.* [38] also found nitrogen concentrations significantly impacted *D. frontalis* fitness. Regions in trees where larvae survived to pupate contained the highest nitrogen concentration, and trees and regions with the highest nitrogen concentrations produced the biggest beetles. Beetle size is strongly correlated with beetle survival, fecundity, pheromone production and dispersal [45,46,47,48,49,50,51,52,53], and thus, is a good indicator of beetle fitness. Interestingly, one mycangial fungus, *Entomocorticium *sp., was superior to another, *Ceratocystiopsis ranaculosus*, at concentrating nitrogen [38]. This difference may explain why *D. frontalis* individuals that develop with *Entomocorticium* are larger and have higher lipid contents than those that develop with *C. ranaculosus* [54], and why beetle populations with a higher prevalence of *Entomocortium* sp. exhibit more rapid population growth [54,55,56].

In contrast to *D. frontalis*, *Ips grandicollis* appears to employ the high consumption rather than the diet modification strategy [38]. These beetles feed extensively in phloem, do not produce feeding chambers, and do not appear to depend on fungi for nutrition, although they do vector ophiostomatoid fungi [57,58]. Although *I. grandicollis* adults are only slightly larger than *D. frontalis* adults, their larvae consumed 79% more phloem than *D. frontalis* larvae [38], supporting the hypothesis that without diet supplementation with fungi, larvae must consume more phloem to meet their nitrogen requirements. Given that *I. grandicollis* is likely to feed at least incidentally on the various fungi it vectors, these results indicate that not all fungi areequally effective as supplements to beetle diets.

Other dietary requirements of the insect macrosymbiont may also influence feeding strategy. For example, insects require sterols for normal growth, metamorphosis, and reproduction. However, insects, unlike most other animals, are unable to synthesize these compounds, and thus, are dependent upon a dietary source [59,60,61]. Sterols are present in plant tissues, but typically only in low concentrations [62], or in forms not usable by insects [59]. For phloem-feeding bark beetles, whose food may contain inadequate concentrations of usable types of sterols, fungal symbionts may provide an alternate source. Fungi typically produce ergosterol, a sterol which is highly usable by many insects [59]. In several insect-fungus symbioses, the insect associate depends on ergosterol production by the fungal associate to meet its sterol requirements [63,64]. This is the case for xyleborine ambrosia beetles [33,65,66] and possibly the coffee berry borer, *H. hampei* [17, but see 67], and may also be true for some bark beetles [68].

The ergosterol contents of ophiostomatoid fungi associated with ambrosia and bark beetles have been investigated for only a few species. For fungi associated with *Xyleborus* ambrosia beetles, ergosterol content ranged from 0.12–0.24% [69]. However, for three species of fungi associated with two *Dendroctonus* bark beetle species, the ergosterol content was much higher at 0.88–1.06% [68], indicating that these fungi may also provide good sources of sterols for their hosts.

For phloeomycophagous bark beetles, the importance and role of fungi in host nutrition may vary by life stage. An experimental study on *D. ponderosae* reported that larvae feed primarily in sterile phloem, and thus do not depend on fungi to complete development [70]. In that study, single pairs of *D. ponderosae* were introduced into logs with ends waxed to retard drying, then held at constant temperatures. Some first instar larvae and all teneral adults were associated with fungi, but intermediate stages of development occurred in sterile phloem. However, in a recent study [71] conducted under field conditions, in naturally infested trees with natural attack densities of beetles (and fungi), approximately two-thirds of 1^st^ instars and 100% of all later instars were located in phloem colonized by fungi. Gut dissections revealed that the symbiotic fungi were ingested by larvae along with their phloem diet. In addition, larvae often migrated back into older portions of the gallery, presumably to feed where the fungi were best established. Such turning behavior by larvae in axenic phloem was also observed by [72], who speculated that such behavior may be linked to the need for larvae to feed in areas containing fungal growth.

Development and feeding on fungus-colonized phloem is common for many bark beetles and has also been observed in other experimental studies [73]. However, not all fungi are equally desirable as food and each association must be considered independently when assessing potential benefits from fungal feeding. For example, *D. frontalis* encountering areas stained by the antagonistic fungus, *O. minus*, turn to avoid feeding in these areas. However, the tunneling behavior of *D. ponderosae* and *I. pini *is unaffected by the presence of staining caused by *G. clavigera* and *O. ips* [73]. Furthermore, in choice tests, *D. ponderosae* larvae chose stained phloem (containing *G.clavigera *and *O. montium*) for feeding significantly more often than unstained phloem [74].

Although Adams & Six [71] found that larvae of *D. ponderosae* are phloeomycophagous, the mere ingestion of fungi does not, by itself, indicate that fungal feeding is beneficial to a developing brood. Unfortunately, the relative intractability of these systems to manipulative experimentation has limited our knowledge of how mycophagy affects host development and fitness. However, studies conducted on two mycangial *Dendroctonus* species in naturally infested material indicate that fungal associates can have a considerable impact on host beetle fitness by affecting larvae. *Dendroctonus frontalis* individuals that develop with mycangial fungi are larger than those that develop without mycangial fungi [38,54,75]. Because adult beetle size is determined by larval nutrition, larger adult size in the fungus-associated beetles cannot be a result of maturation feeding on spore layers by teneral adults [76]. Furthermore, larval survival is higher, and feeding galleries of *Dendroctonus* are shorter, in the presence of mutualistic fungi than in their absence, indicating that fungus-colonized tissues have higher nutritional contents [38,75,77]. Not surprisingly, the multiple fungal partners associated with a host tend to vary in their effects on beetle broods. For *D. frontalis*, *Entomocorticium* sp. supports higher host survival and larger body size than does *C. ranaculosus*. For *D. ponderosae*, *G. clavigera* supports faster brood development and higher brood production than does *O. montium* [77]. Similar results were found in an experiment conducted with a non-mycangial beetle, *I. paraconfusus*. Axenically reared beetles, and those reared with the antagonistic fungus *O. minus*, were smaller than beetles reared with symbiotic fungi associated with the beetle, and larval tunnels were significantly longer when larvae were associated with *O. minus* than when not associated with fungi [72].

The role of mycophagy in adult nutrition is poorly understood. Teneral adults of mycangial bark beetles feed on dense layers of spores that grow on the pupal chamber walls, before emerging to disperse to new host trees (Figure 3) [70,77]. This also may be true for several non-mycangial beetles that are consistently associated with fungi that produce spore layers in their pupal chambers. This period of feeding on spores as new adults may be important for beetles to acquire fungi in their mycangia and/or on their exoskeletons for dispersal to the next host tree and the next generation of beetles. However, feeding on spores at this time also appears to be important in adult reproduction. New adults of *D. ponderosae* that did not feed on the conidia of mutualistic fungi (*G. clavigera*, *O. montium*), tunneled and fed extensively in phloem. In contrast, insects that fed on spores did not tunnel and feed in phloem and emerged very close to the pupal chamber [74]. New *D. ponderosae* adults that did not feed on spores had very high rates of rejection of logs, produced few galleries, and did not produce broods. In contrast, new adults that fed on spores of either of the beetle’s symbiotic fungi tended not to reject logs, usually produced galleries, and many also produced broods [77]. Axenic *I. paraconfusus *adults also did not oviposit, while those associated with fungi did [72]. These results indicate that feeding on fungal spores by new adults may be critical for adult nutrition and reproduction for at least some bark beetle species.

Obligate symbiosis is typically defined as the inability of one or both interacting partners to live without the other. At its simplest, this can mean that if, in a single reproductive cycle of a partner pair, one partner is removed, the other partner dies or cannot reproduce. However, the term can also denote partnerships where the separation of host and symbiont results in fitness costs that, over only a few generations, eventually result in the loss of one or both partners. Determining whether a particular symbiosis is obligate can be an immensely difficult task. It is challenging, and sometimes impossible, to produce aposymbiotic hosts. Furthermore, the processes used to remove symbionts can be extremely stressful to hosts, bringing into question the validity of experiments conducted with such hosts. A challenge in testing for dependence is that hosts must be reared at least through the F2 generation to control for maternal effects [66,78]. For insects such as bark beetles that can be difficult to rear through the F1 generation, this is a serious obstacle. To date, obligacy has been shown (and looked for) in only a few bark beetle-fungus symbioses [56,77]. No studies that claimed to successfully rear beetles without symbiotic fungi meet stringent requirements for testing for dependence on symbiotic fungi for nutritional supplementation, either because they were conducted only through the F1 generation [72,79], or because the beetle’s diet was supplemented or contaminated with fungi or fungal products [80,81,82].

For bark beetles, detecting obligacy can be further complicated by multipartite associations involving hosts with two, less often three, consistent fungal associates. In some associations, these symbionts may provide a similar benefit to the host (symbiont redundancy) [56,77]. In such cases, the host may be dependent on the presence of a symbiont, but not any one symbiont, in particular. The concept of ecological (or functional) redundancy has been particularly well-developed in the field of biodiversity conservation, but much less so in symbiology, where most efforts have focused on pollinator assemblages [83]. The concept of symbiont redundancy is further developed for bark beetle-fungus symbioses in a later section.

To this point, I have focused primarily on fungi as mutualists of bark beetles. However, many ophiostomatoid fungi are inconsistently associated with particular beetle species and often are associated with several beetle species across a wide geographic area (ex. *O. piceae*, *O. penicilliatum*). Such broadly distributed fungi are probably opportunistic commensals, benefiting from transport, but without significant reciprocal effects on the host [7,10]. Other fungi in this group are antagonists and their presence results in lowered host fitness. For example, *D. frontalis* developing in areas colonized by *O. minus *seldom survive (Figure 3) [84,85]. Why some ophiostomatoid fungi are beneficial while others are antagonistic, or have no apparent effect on their host, is unknown, but may reflect their ability to concentrate nitrogen [38], to produce adequate amounts of sterols [68], or to produce toxic metabolites [86].

Our ability to make generalizations about bark beetle-fungus symbioses is constrained by a lack of knowledge on all but a very few systems. Only a few studies have been conducted and the majority of these have focused on the tree-killing, economically important beetles. This focus on aggressive beetles has yielded a highly biased view of bark beetle-fungus interactions, including a near exclusive focus for many years on the potential, and still unsubstantiated, role of the symbiotic fungi in tree-killing [12]. However, in the Scolytinae, tree-killing is actually a relatively rare event of life history. Instead, most scolytines are restricted to weak, dying, or more often, recently killed trees. For example, of the hundreds of scolytine species in North America, only 7–10 commonly kill trees [14]. The majority of the remaining non-tree-killing species are associated with fungi in one way or another, but remain mostly unstudied.

## 2. Evolution of Scolytinae-Fungus Symbioses

The Scolytinae are thought to have arisen in the Late Jurassic or Early Cretaceous periods, with the most recent estimates dating to about 100 million years ago [87,88,89]. Conifers are probably the ancestral hosts of the Scolytinae and its most closely related subfamilies in the Curculionidae [90,91]. The putative sister group to these subfamilies, the Derolominae, is associated with monocots, implying that a common ancestor shifted from angiosperms to conifers [91]. In the Scolytinae, this switch was followed by several returns to angiosperms, then several subsequent reversals to conifers. Each shift to angiosperms was accompanied by increased species diversity, whereas reversals to conifers resulted in low diversity [91].

Ophiostomatoid fungi apparently arose 200 million years ago [92], with the groups containing *Ophiostoma* (and allied genera) and *Ceratocystis* probably diverging around 170 million years ago [91]. Therefore, these fungi predate the Scolytinae and may have evolved adaptations for insect dispersal prior to their association with scolytine beetles. They were probably originally vectored by other arthropods, possibly including weevil ancestors of the Scolytinae [91].

The ambrosia and bark beetles do not form exclusive monophyletic groups within the Scolytinae; rather, the two fungal feeding strategies evolved several times independently. The origins of ambrosia feeding all followed shifts to angiosperms, although there apparently were reversals to conifer feeding by some temperate ambrosia beetles [88]. The ambrosial feeding habit has evolved at least eight times (possibly more) from different beetle tribes containing phloem-feeding beetles associated with *Ophiostoma*, *Grosmannia*, and/or *Ceratocystiopsis* species [91,93]. These ambrosial feeding strategies have been estimated to have evolved 21–60 million years ago, depending on beetle lineage. Likewise, within the Scolytinae, phloeomycophagous bark beetles occur in several dispersed tribes, ranging from the Tomicini to the Ipini [91].

The paraphyletic nature of the ambrosia beetle-associated genera, *Ambrosiella* and *Raffaelea*, with derivations from both *Ophiostoma* and *Ceratocystis*, may reflect these multiple origins and host shifts. When some beetles switched to angiosperms, some apparently maintained associations with *Ophiostoma*. Others may have switched to *Ceratocystis*, which they may have encountered for the first time in their new hosts. *Ceratocystis* species have morphological adaptations for insect dissemination similar to those of *Ophiostoma*, and may have been pre-adapted for vector relationships with these beetles. If some *Ceratocystis* species also provided nutritional benefits, then once associations formed, similar lifestyles may have led to a convergence of form in the fungi, and to the multiply derived genera that are evident today. The modern association of *Ceratocystis* species with a very few conifer-using bark beetles may indicate that some fungi ‘followed’ beetles back to conifers. Interestingly, at least one lineage of *Ambrosiella *(now transferred to *Hyalorhinocladiella*) is not associated with ambrosia beetles, but rather with species of *Ips*, *Polygraphus*, and *Hylurgops* [30], indicating an independent origin of this morphological form with bark beetles in conifers. Past reliance of fungal taxonomy on morphology has led to the current unnatural classification used for many fungi associated with Scolytinae. In many cases, convergent evolution for an insect-adapted lifestyle has led to similar forms resulting in unrelated fungi being placed within the same genus. Rigorous revisions of these genera to better reflect actual relationships will vastly improve our understanding of these fungi and how interactions with scolytine hosts ultimately influence their form, function, and distribution.

Floristic composition and diversity may be important drivers of diversity in herbivorous insects [94,95]. Indeed, enhanced rates of diversification in angiosperm-feeding beetle lineages resulted in nearly half of the species in the order Coleoptera, which contains much of the insect biodiversity on Earth [95]. However, for many insects, including the Scolytinae, host plant diversity may be only one of several factors influencing diversification. For ambrosia beetles, the adoption of a strictly mycophagous habit may have led to extensive species radiations in the Xyleborini and the Platypodini [96]. However, these radiations occurred mainly in tropical rainforests, where both warm temperatures and high humidity favor fungal growth [97] and the diversity of trees is very high, confounding our ability to detect drivers of diversity in these systems. In addition, the massive radiation of the Xyleborini occurred simultaneously with the development of inbreeding. Because, in several lineages of ambrosia beetles, the development of a strictly fungus-feeding lifestyle did not result in extensive radiations and they remain relatively species poor, we cannot conclude that the development of strict fungal feeding in and of itself supports radiation. However, while conifer-using lineages within the Scolytinae are species-poor, relative to some angiosperm-using lineages, three tribes [Tomicini (including the Hylastini), Ipini, and Corthylini] are among the most species-rich conifer associations known [91] indicating that fungus feeding may, at least at times, support greater species diversity.

The development of mutualisms with fungi also may have supported the diversification of scolytine lineages inhabiting conifers. Two of the three most diverse conifer-using tribes in the Scolytinae, the Tomicini and the Ipini, contain most known examples of mycophagous bark beetles. The third tribe, the Corthylini (which also contains an ambrosia beetle lineage), contains the species-rich Pityophthorina, many of which use conifers, and which are also associated with fungi [98,99,100], but remain uninvestigated for mycophagy.

Mutualism allows organisms to excel in marginal habitats, exploit new niches, avoid competition, and buffer environmental variability [11,101]. In the cases of both ambrosia beetles and mycophloeophagous (combined fungus-phloem feeders) bark beetles, the use of fungi for food has expanded the capacity of these insects to use nutrient-poor plant resources [15]. Nutrition/transport-based mutualisms evolved many times in the Scolytinae and transitions from a strictly plant-based diet to a combined or strictly fungus-feeding strategy perhaps evolved relatively rapidly. The evolution of mycangia may be a particularly useful metric of both the advantage, and the rapidity, of the evolution of fungus-beetle mutualisms. Mycangia evolved independently many times in the Scolytinae. They are present in almost all ambrosia beetle species and in many bark beetles. Furthermore, mycangia within the same genus occur in different body regions, or differ in their distribution between the sexes, indicating independent, rapid origins over a very short evolutionary time frame [91,102]. Mycangia occur across many beetle tribes, including some basal groups, suggesting that fungus feeding has been advantageous to the Scolytinae from its origin.

The propensity of Scolytinae to form nutrition/transport-based mutualisms with fungi is probably linked to two characteristics that have exemplified the subfamily since its beginnings: the exploitation of inner plant tissues and the formation of associations with fungi that grow there. However, the exact path leading to the formation of these mutualisms is unknown. Two models for evolutionary transitions from a plant-based diet to ‘fungiculture’ in insects have been proposed [103]. In the ‘transmission first’ model, the insect is first associated with a fungus as a vector, then begins to obtain nutrition from the fungus, and finally relies on the fungus as a food source [11,103]. In the ‘consumption first’ model, an insect lineage begins to incorporate fungi into a generalized diet and then becomes a specialized fungivore. Implicit in the second model is that both insect and fungus must also develop adaptations for vectoring to ensure transmission from generation to generation. Both models are tenable for the Scolytinae, and it is likely that various forms of both models have occurred to produce the associations that we see today.

If current day associations with fungi reflect phylogenetic history, then scolytine beetles were associated with *Ophiostoma* (and allied genera) from their origin. Many, if not all, of the most primitive members of this subfamily (ex. *Hylurgops*, *Hylastes*, *Pseudohylesinus*) vector *Grosmannia* and *Ophiostoma *species, but with no evident benefit to the host. Such apparently strictly vector associations occur throughout the Scolytinae (ex. *Scolytus*, *Orthotomicus*), interspersed between the phloeomycophagous bark beetle and ambrosia beetle lineages. Such vector associations are unsurprising, given that ophiostomatoid fungi are very well adapted to insect dispersal [104] and that these adaptations appear to have arisen prior to the origins of the Scolytinae [91]. In addition, both beetles and their associated fungi colonize early in succession, colonizing living (at least in the initial stages of attack) or freshly killed plant material. As a consequence, both must arrive very early in the colonization sequence. Among the many loose associations that formed, some eventually developed into nutrition/transport-based mutualisms of the ambrosial type with beetles exploiting angiosperms, and of the phloeomycophagous type for beetles exploiting conifers. Of note is that while some ambrosia beetles attack conifers, there are no known phloeomycophagous species among the bark beetles that colonize angiosperms.

Regardless of how these associations originated, it appears that once established, reversals from the fungus-feeding state are rare or nonexistent. No reversals to a non-ambrosia feeding state are known in ambrosia beetles [91] or for other insect-fungus nutrition/transport-based mutualisms, including the fungus-gardening ants and termites [11]. This indicates that the transition to obligate mycophagy is a major and potentially irreversible change that constrains subsequent evolution [11]. Even where beetles have lost the capacity to vector the fungi, they continue to exploit fungi through mycocleptism [105].

The independent evolution of fungus feeding many times in the Scolytinae suggests that an overall tight concordance of phylogenies of the beetles and their fungal associates should not be expected. However, for particular lineages of beetles, especially those with shared mycangial types and common obligate associations with fungi, we might expect evidence of tightly linked evolutionary histories and cospeciation. This has not been explicitly investigated, except in one study where it was found that some *Ceratocystiopsis* and *Dendroctonus* possessing pronotal mycangia, and some *Grosmannia* and *Dendroctonus* possessing maxillary mycangia, show evidence of cospeciation [102]. However, the same study revealed that host switching and/or colonization events also occurred in these same associations. While no other studies looking explicitly for cospeciation have been conducted in the Scolytinae, the distribution of fungal species among various host beetles indicates that host switching has been common, even among ambrosia beetle lineages and their fungal associates [7,28,91].

There are several reasons why strict cospeciation of beetle hosts and fungal symbionts may be rare, or at least difficult to detect, in the Scolytinae. Two factors appear to greatly facilitate cospeciation: strict vertical transmission of symbionts, and restricted options to acquire hosts or symbionts from outside the relationship [106,107]. Neither criterion appears to be strictly met by scolytine-fungus associations. The presence of highly specific organs to transmit symbionts (mycangia) at first may seem to indicate strict vertical transmission. However, unlike endosymbioses with symbionts transmitted directly from mother to offspring via the egg, in scolytine-fungus ectosymbioses, the fungi are inoculated by the beetles into plant tissues where they grow for a period of time independent of the host before being reacquired by offspring as teneral adults. This period of growth in wood presents a weak link in the transmission process and provides an opportunity for horizontal transmission of symbionts.

Vertical transmission may be more reliable in some ambrosial systems than in others, and more reliable in ambrosial systems than in phloeomycophagous systems. For example, in ambrosial species of the Xyleborini, only females possess mycangia, and mating occurs between siblings in the natal substrate [108,109,110]. For these beetles, males do not disperse and only females contribute inoculum to the brood. However, for some ambrosia and most bark beetles, both sexes disperse to, and mate in, new substrates prior to initiating a brood [108]. For these insects, both sexes carry fungi to the breeding substrate, greatly decreasing the likelihood of strict vertical transmission. This is true regardless of whether one or both sexes, or neither sex, possess mycangia. For mycangial beetles, one or both parents may transmit mycangial fungi not only in mycangia but also on their exoskeletons (although mycangial fungi are often transmitted at much lower rates on the exoskeleton than in mycangia, [111]). For non-mycangial beetles, fungi are transported on the exoskeleton, although efficacy of vectoring can vary by sex [112]. Very importantly, parents often originate from different broods and often from different trees. This means that the fungi that each contributes to its offspring may be different species or different genotypes of the same species.

For both ambrosia and bark beetles, this is further complicated because commensal ophiostomatoid fungi are often also transported by parents. Multiple scolytine beetle species (and their fungal associates) often cohabit one tree, further increasing the potential pool of fungi that a brood might contact. Therefore, even if a beetle begins development with one fungus faithfully transmitted by only one parent, it is liable to be exposed to, and potentially acquire, several other fungi by adulthood. Such exposure, over time, may result in host switching or colonization events. It may also account for the multipartite nature of many of these associations. The ability of hosts to occasionally acquire new partners might have led, not only to the replacement of old associates with new, but also to the addition of new associates to old. In some cases, new associates may be acquired because of their superior qualities. In contrast, some symbionts may be ‘cheaters’ that have infiltrated established associations between the host and superior, established symbionts.

However, despite evidence that host switching and colonization events were common over evolutionary time, many mutualistic beetle-fungus symbioses are highly specific. This indicates that host switching is constrained, and that mechanisms exist to ensure fidelity of partners. In contrast, associations of fungi with beetles that merely act as vectors are less constrained. This may explain why some beetles easily acquire novel ophiostomatoid species when introduced into new habitats or when new fungi are introduced into their native range by exotic beetles or in wood [100,113,114].

Abiotic factors may also greatly affect acquisition of fungal associates by beetles, and thus may also act to disrupt vertical transmission. As a season progresses, variation in environmental conditions can cause variability in the relative growth rates of fungal symbionts. This influences which fungi sporulate in the pupal chamber at the time of teneral adult maturation feeding, and thus determines which fungi are acquired by the beetles and dispersed to the next host plant and the next generation of beetles [115] (discussed further in a later section). Therefore, as environmental conditions vary over a season, over years, and by location, fungal assemblages associated with a beetle species may vary and shift merely by the influence of abiotic factors. Indeed, the abiotic environment has played, and continues to play, an important role in determining the distribution of the fungi with beetle hosts on both local and regional scales.

Absence of evidence of strict cospeciation does not imply that these are wholly unconstrained associations. Cospeciation and host switching/colonization events represent phylogenetically- and ecologically-mediated evolutionary processes [116]. These processes, while seemingly independent, can be coupled, with phylogenetic relationships strongly influencing the nature of a host shift that is otherwise ecologically mediated [116]. In the case of scolytines and ophiostomatoid fungi, host shifts occur, but usually to phylogenetic relatives (within Ophiostomatales or Microascales), although often not to a sister species. Therefore, while we see little evidence of cospeciation, host transfers appear to be mostly constrained to members within the Scolytinae and the ophiostomatoid fungi.

Phylogenetic conservatism, however, has not been absolute between ophiostomatoid fungi and scolytines. For example, *D. frontalis* possesses two mycangial fungi. One, *Entomocorticium* sp. A, is a Basidiomycete. This fungus appears to be a superior symbiont compared with the more coevolved ophiostomatoid associate, *C. ranaculosus*, indicating that this Basidiomycete was acquired opportunistically because of its benefit to the host. Furthermore, ophiostomatoid fungi can also be consistently found in *Protea* infructescences, in soil, and even in the mounds of fungus-gardening termites [117,118]. These ophiostomatoid fungi in Proteas and termite mounds lie in a highly-derived clade within *Ophiostoma* and thus these associations probably formed after those between *Ophiostoma* and bark beetles [18]. Therefore, while phylogenetic conservatism clearly has imposed constraints, new opportunities have been exploited, resulting in the formation of associations between beetles and non-ophiostomatoid fungi and ophiostomatoid fungi and non-scolytine hosts.

## 3. The Role of Biotic and Abiotic Factors in Shaping Scolytinae-Fungus Symbioses

The structure of biological communities is seldom determined by a single major factor or process, but by many independent and interacting processes. This is also true for subsets of interactions within the broader community including symbioses. Below, I discuss the major biotic and abiotic factors and processes that influence the structure of symbiotic fungal assemblages associated with bark beetles.

### 3.1. The Host Plant

The host plant provides the substrate and nutritional resources that support the growth and reproduction of both beetles and fungi. The majority of scolytines and their associated fungi colonize freshly killed plant material (whether the beetles themselves kill the plant or arrive after the fact), which means that, at least initially, the plant is a relatively inhospitable environment. Host tree defenses present at the time of colonization [119] can repel or even kill host beetles and are often fungitoxic or fungistatic. Aggressive beetles reduce host tree effects by a pheromone-mediated mass attack that kills the tree and quickly reduces tree defenses [120]. Fungal associates are often pathogenic to the host plant, facilitating their survival in still living or newly-killed plant tissues until defenses subside. Interestingly, most fungi associated with tree-killing beetles (primary and secondary, e.g., *D. frontalis*, *I. pini*) possess relatively low levels of virulence [6,10]. In contrast, fungi associated with beetles that develop in living trees, where the tree does not die (e.g., *Hylurgops*, *Hylastes*, *D. valens*, *D. terebrans*), possess relatively high levels of virulence [121,122,123]. These differences in virulence may reflect differences in fungal life histories. For fungi associated with tree-killing beetles, high levels of virulence are unnecessary because plant defenses are active only briefly. On the other hand, fungi associated with beetles developing in living hosts may require greater virulence to avoid containment and to be able to persist in a continuously defensive tree until new brood adults disperse up to a year after initial introduction.

The challenge of using trees as substrate does not end once defenses have abated. The quality and condition of a host tree changes, often radically, over the development period of the beetles. Tree tissues are highest in nutrients and moisture at the time of colonization, but by the time of brood adult emergence and dispersal, much of the phloem resource has either been consumed or has become badly degraded and depleted of nutrients [8]. Furthermore, moisture loss over this period can be considerable, often contributing to the mortality of substantial numbers of the beetle brood and contributing to decreasing areas in the tree colonized by symbiotic fungi [124,125].

Changes in chemistry, moisture and nutritional content of the host plant can affect the distribution and relative prevalence of fungal associates within a tree. Adams and Six [71] observed that the relative prevalence of *G. clavigera* and *O. montium* (the former a moderately virulent pathogen, the latter a weak pathogen/saprobe) associated with *D. ponderosae *shifted dramatically over beetle development. These shifts were probably driven by changes in tree defenses and moisture conditions (and temperature, discussed below). Variation in virulence among fungal associates affects the rate and timing of their capture of resources within the tree. Initially, fungi with greater virulence (and typically greater tolerance to high levels of moisture and low levels of oxygen) grow more rapidly and capture more resource [126]. However, as defenses decline and tree tissues begin to dry, the less virulent, more saprophytic fungi, begin to dominate. Furthermore, while some fungi are highly competitive in one set of conditions, they may be poor competitors under others [125]. Thus, changes over time within the tree influence not only relative rates of growth and primary resource capture, but also the outcome of direct competition among the various fungi [125,127].

### 3.2. Microbes

Bark beetles and their symbiotic fungi coexist with a multitude of microbes. These include yeasts and bacteria that colonize beetle galleries and that are likely vectored into the tree by the beetles, and endophytic bacteria and fungi that grow within host tree tissues irrespective of the presence of the beetles. While most studies conducted on microbes associated with beetle galleries are surveys [128 and others], only a few have focused on the potential ecological roles of these microbes in these microhabitats [129,130,131,132,133]. Nair *et al.* [134] isolated a bacterium, *Bacillus mojavensis*, from galleries of the ambrosia beetle, *Xylosandrus compactus*,that inhibited several fungi, including the ambrosial fungus of the beetle. Adams *et al.* [135] found that both yeasts and bacteria have substantial effects on the growth of the two mycangial fungi of *D. ponderosae*. The yield of *O. montium* grown *in vitro *individually with two yeasts and a bacterium isolated from larval galleries was much greater than the yield of *O. montium* grown alone. However, the relative yield of *G. clavigera* grown with these same microbes was less than when it was grown alone. These results suggest that at least some microbes found in larval galleries facilitate the growth of *O. montium *and are antagonistic to *G. clavigera*. A bacterium isolated from uncolonized phloem (a putative endophyte) strongly inhibited relative yield of both *G. clavigera* and *O. montium* and appears to be an antagonist to both. Subsequent work has characterized various effects of bacteria associated with bark beetles on symbiotic fungi indicating they may, at least in part, mediate interactions between the symbiotic fungi and the host beetle [136].

Cardoza *et al.* [132] observed *D. rufipennis* producing oral secretions that inhibited the growth of fungi associated with the host beetle. These oral secretions contained bacteria that inhibited one or more of the fungi, including the ophiostomatoid symbiont, *L. abietinum*. Further, actinomycetes in mycangia may provide some protection to beneficial fungi from antagonistic ones [137].

Work on bark beetle gut communities indicates a high diversity of microbes associated with this niche; however, the roles of these microbes and their potential interactions with bark beetle symbiotic fungi remain poorly understood [138,139]. Overall, it appears that at least some co-occurring microbes impact the distribution of symbiotic fungi through antagonistic or facilitative interactions, with potentially important indirect effects on the fitness of host beetles.

### 3.3. Arthropods

Bark beetles and their symbiotic fungi also share trees with many arthropods. These arthropods include natural enemies (predators and parasitoids), phloem and wood borers, and fungivores, as well as other bark beetle species. Some of these arthropods significantly affect beetle-fungus symbioses. Bark beetle species that cohabit the same tree can compete for resources. Their fungi may also compete for space and resources while also disrupting contact between a beetle and its normal fungal assemblage.

Some mites, phoretic on bark beetles, have close symbioses with ophiostomatoid fungi [140,141]. These mites feed on their associated fungi and vector them in sporothecae, the structures of their exoskeletons being analogous to bark beetle mycangia. Mites and their associates can have profound effects on the fitness and population dynamics of bark beetles and their associated fungi [141]. Interestingly, a mite-scarab beetle-ophiostomatoid fungus interaction recently reported from *Protea *infructescences [116] indicates that such complex associations involving mites are not limited to bark beetle systems.

Some natural enemies of bark beetles also interact, at least indirectly, with bark beetle-associated fungi. In the *Ips pini—O. ips* and the *D. ponderosae-O. montium-G. clavigera *systems, parasitoids are attracted to fungus-colonized tree tissues and apparently use fungus-produced volatiles for locating beetle larvae and pupae [142,143]. In contrast, in the *D. frontalis*-fungus symbiosis, fungi were not required for attraction to occur [144]. Whether such exploitation of fungal symbionts by parasitoids to locate hosts affects beetle or fungal fitness or population dynamics is unknown.

### 3.4. Temperature

Fungi are extremely sensitive to temperature and most species grow only within a relatively narrow range of temperatures. Optimal growth temperatures and ranges of temperatures supporting growth vary substantially among species. Such differences can greatly affect the distribution of fungi, their relative prevalence, and the outcome of competitive interactions when fungi occur together in a substrate.

For example, Six and Bentz [115] found that temperature plays a key role in determining the relative abundance of the two symbiotic fungi associated with dispersing *D. ponderosae*. The two fungi possess different optimal growth temperatures. When temperatures are relatively warm, *O. montium* is dispersed by new adult beetles, but when temperatures are cool, *G. clavigera* is dispersed. Shifts in the prevalence of the two fungi probably reflect the effects of temperature on sporulation in pupal chambers when brood adults eclose, begin to feed, and pack their mycangia with spores. The two fungi are not highly antagonistic to one another when grown in culture [145] and are often observed or isolated together from phloem or from the same pupal chamber [71,146,147]. The ability of these species to intermingle in tree substrates, and the rarity of fungus-free dispersing beetles, indicates that both fungi are probably present in many pupal chambers, but that depending upon temperature, typically only one will sporulate and be acquired in mycangia at a particular point in time. This determines which fungus is dispersed to the next tree and the next generation of beetles, with substantial implications for the fitness of both beetles and fungi.

Significant effects of temperature on interactions between *D. frontalis* and its two mycangial fungi, and an antagonistic phoretic fungus (associated with mites phoretic on *D. frontalis*) were also observed. The relative abundance of the two mycangial fungi of *D. frontalis* changes seasonally, with *Entomocorticium* sp. A prevailing in winter and *C. ranaculosus* in summer [84]. Their relative frequency was significantly affected by temperature. Increased temperatures probably decreases beetle reproduction directly through effects on the physiology of progeny and indirectly through effects on mycangial fungi. *Entomocorticium* performs poorly at higher temperatures while *C. ranaculosus *is unaffected.

## 4. Stability and Redundancy in Multipartite Systems

Symbioses, particularly mutualisms, are predicted to be inherently unstable and prone to erosion because of cheating by established symbionts or invasion by exploiters [148]. This may be especially true for multipartite symbioses, such as most bark beetle-fungus symbioses, where interactions among symbionts may also affect stability. Many fungal associates of bark beetles are phylogenetically related and have similar life histories. They are introduced into trees by the host beetle, are thought to use the same resources within the tree, and potentially compete for the same space, and ultimately, for the same host beetles when it comes time for dispersal. Thus, the multiple fungal associates of beetle species appear to occupy essentially the same niche. This should result in strong direct competition among symbionts, leading to replacement of weaker competitors by stronger competitors. Moreover, for mutualisms, different symbionts, being different organisms, are not expected to provide exactly the same degree of benefit to the host. Therefore, symbionts that provide inferior benefits should be selected against, and superior symbionts should move toward fixation with the host. Despite these predictions, many multiple-partner associations have apparently been relatively stable for long periods of evolutionary time [102], indicating the existence of factors or mechanisms that contribute to their stability.

Questions of how and why a host maintains two or more mutualistic symbionts are particularly interesting. At first glance, inferior symbionts appear to be inherently detrimental to the host because they displace the more beneficial symbiont(s) from a proportion of the host population. This should lower the fitness of individual hosts relative to those with superior symbionts. This may be especially important for aggressive beetle species that mass attack trees, and whose success ultimately is linked to host population size.

When considering which symbionts are superior, it is important to remember that roles and intensities of effects vary with environmental conditions. Environmental heterogeneity is a fundamental attribute of biological communities [149], and the function of any given species can vary considerably across natural gradients, both within a community and among different communities [150]. This variability in function as conditions change has been called ‘context dependency’ [151]. Gradients of temperature, moisture, and other environmental variables comprise the essential axes of species’ ecological niches and these factors exert major influences on the ecological performance of organisms in nature [152]. Within the geographic range of an organism, some conditions will be more suitable for survival growth and reproduction. This means that some symbionts that are ecologically extraneous (or inferior) at one point on a multifactoral environmental gradient may be essential (or superior) at another.

Symbionts associated with a beetle can appear to occupy a common niche when in actuality the niches may differ greatly. Each partner in these symbioses responds differently to the same set of environmental gradients. This may translate to relatively large differences in the effectiveness of different symbiont genotypes (different species or strains of one species) under different environmental conditions. Furthermore, if shifts in the environment are unpredictable or rapid relative to the generation time of the host, then host specialization on one symbiont may not be favored. Under such circumstances, multiple symbionts may be advantageous, because they increase the chance that at least one symbiont partner is effective under any prevailing set of environmental conditions.

For example, as reviewed above, the two fungi associated with *D. ponderosae* possess different temperature tolerances [115,153,154]. These differences determine which fungus is vectored by dispersing host beetles as temperatures fluctuate over a season. This temperature-driven symbiont shifting may provide a mechanism that has allowed both fungi to persist in a long-term symbiosis with their host. By growing at different temperatures, and thus at different times, the fungi minimize competition with one another except at a narrow range of temperatures where the growth of both fungi is equally supported. In turn, the beetle may benefit by reducing its risk of being ‘left alone’ by exploiting not one, but two symbionts, whose combined growth optima span a wide range of environmental conditions. For bark beetles, such as *D. ponderosae*, which inhabit a broad geographic range and highly variable habitats, possessing multiple symbionts may be especially important.

It may be useful to view multipartite symbioses from the perspective of functional redundancy. The idea that many species in ecosystems perform the same or very similar functions (members of a functional group) has been used extensively in conservation theory [155]. The concept of functional redundancy suggests that the presence of a diversity of functionally equivalent species enhances the resilience of an ecosystem and its ability to function after perturbation [155]. This concept may also be applicable to symbioses, especially ectosymbioses, where hosts often have multiple symbionts that fulfill similar roles (symbiont redundancy) and where both partners are exposed to vagaries of the environment. Symbiont redundancy may contribute to resilience and help maintain functions in symbioses that occur in variable habitats where one symbiont alone may not suffice. Symbionts in the same ‘functional group’ may be redundant in the resources provided to a host, but possess different responses along environmental gradients, allowing the symbiont community as a whole to respond to changes in the environment that occur both seasonally and from year to year.

## 5. Conclusions and Future Directions

Symbioses between Scolytinae and fungi are complex, varied and still poorly understood. While our understanding of these systems remains rudimentary, the recent revival of interest in them has led to a rapid accumulation of information. Molecular taxonomic tools have enabled researchers to accurately identify fungal partners and to resolve phylogenetic relationships of beetles and fungi alike. This renaissance emerged because of the willingness of investigators to test new paradigms and to apply ecological and evolutionary theory to these interactions. Because of this, the near future should be a very exciting period, moving us rapidly toward an integrated understanding of how these organisms interact with each other and the environment, revealing how their interactions have developed and been maintained over time.
